# Altered expression of microRNAs in the myocardium of rats with acute myocardial infarction

**DOI:** 10.1186/1471-2261-10-11

**Published:** 2010-03-01

**Authors:** Bing Shi, Yanhong Guo, Juan Wang, Wei Gao

**Affiliations:** 1 Department of Cardiology, Peking University Third Hospital, Beijing, China; 2 Department of Cardiology, Beijing Military General Hospital, Beijing, China; 3 MOE key Laboratory of Molecular Cardiology, Peking University, Beijing, China; 4 Department of Biomedical Informatics, Peking University Health Science Center, Beijing, China

## Abstract

**Background:**

MicroRNAs(miRNAs) are important cellular components and their dysfunction is associated with various diseases. Acute myocardial infarction (AMI) is one of the most serious cardiovascular diseases. Although several miRNAs are reported to be associated with AMI, more novel miRNAs are needed to further investigate and improve certainty

**Methods:**

We applied a well-established acute myocardial infarction rat model and performed miRNAs microarray experiments upon the myocardium tissue of rats with AMI and under sham control. We identified the differentially expressed miRNAs and analyzed the function of miRNA targets, transcription factors, and host genes based on bioinformatics.

**Results:**

As a result, the levels of expression of seventeen miRNAs significantly deregulated, of which four miRNAs were further validated by qRT-PCR. In addition, we observed that the transcription factors, targets, and host genes of these deregulated miRNAs are enriched in cardiovascular-related functions.

**Conclusion:**

We found that the miRNAs expression level altered in rats with AMI and differentially expressed miRNAs may be novel biomarkers of AMI.

## Background

MicroRNAs (miRNAs) are small non-coding RNAs that represent one of the most important cellular components. Increasing evidence indicates that miRNAs have distinct expression profiles and play crucial roles in the regulation of gene expression during development stages such as cell growth, differentiation, and apoptosis[1]. Deregulation of miRNAs is associated with various diseases including cardiovascular diseases [1-4]. In recent years, efforts have been made to identify the miRNAs associated with cardiovascular diseases [1]. For example, miRNAs involved in cardiac hypertrophy and heart failure such as miR-208, miR-133, miR-195, miR-21, and miR-126 have been reported in several studies [5-8].

Acute myocardial infarction (AMI) is one of the most serious cardiovascular diseases [9]. Recently, some miRNAs for example miR-21, miR-1, miR-216[10], and miR-29 family[11], were reported to be deregulated in myocardial infarction. However, considering the complex genetic variants in AMI, further investigation is needed to ascertain if more novel miRNAs are associated with AMI.

In this study, to explore the expression of miRNA in ischemic myocardium in rat with AMI, we employed microarray technique on the border zone myocardium of rats with AMI and sham-operated controls. As a result, we found that the levels of expression of seventeen miRNAs significantly changed in the progression of AMI. Furthermore, some of the deregulated miRNAs were confirmed by qRT-PCR, and the functions of miRNA targets, transcription factors, and host genes were also analyzed.

## Methods

### A rat model of AMI

We utilized an *in vivo *rat model to study altered expression of miRNA in the border zone myocardium of rats with AMI. Experimental acute myocardial infarction models were induced by the ligation of anterior descending coronary artery in adult Spraque-Dawley rats (180 to 200 gram), as described by Shyu et al. [12]. On the first postoperative day, the survivals were randomly divided into three groups: 2 days after AMI, 7 days after AMI, 14 days after AMI. In the sham-control group, rats were exposed to all surgical procedures except the ligation of the anterior descending coronary artery. Each group has five individual samples. This study was approved by the Institutional Laboratory Animal Care and Use Committee of Peking University Health Science Center (Peking, China). The investigation conformed to the Guide for the Care and Use of Laboratory Animals published by the China National Institute of Health.

### Microarray analysis

For miRNAs microarray analysis, total RNA from the myocardial tissues in the border zone of infracted myocardium was isolated using Trizol reagent (Invitrogen) according to the manufacturer's instructions. Low-molecular-weight RNA (<200 nt) was isolated from the 30 μg total RNA using a mirVana miRNA Isolation Kit (Ambion, Austin, TX, USA). Profiling of miRNAs expression was analyzed by CapitalBio (CapitalBio Corp, Peking, China) using CapitalBio Mammalian miRNA Array V3.0.(Which includes 924 known miRNAs for human, mouse and rat). The miRNA microarray from CapitalBio Corporation was a double-channel fluorescent chip. Experimental procedures were performed as described in detail on the website of CapitalBio http://www.capitalbio.com. The T4 RNA ligase labeling method according to Thomson's protocol was adopted[13]. Briefly, 4 ug of low-molecular-weight RNA was labeled with 500 ng 5'-phosphate-cytidyl-uridyl-cy3/5-3' (Dharmacon) with 2 units T4 RNA ligase (NEB). The labeling reaction was performed at 4°C for 2 hours. Labeled RNA was precipitated with 0.3 M sodium acetate, 2.5 volumes ethanol and resuspended in 15 μl of hybridization buffer containing 3× SSC, 0.2% SDS and 15% formamide. The array was hybridized at 42°C overnight and washed with two consecutive solutions (0.2% SDS, 2× SSC at 42°C for 5 min, and 0.2% SSC for 5 min at room temperature). The miRNAs Arrays were scanned with a confocal LuxScan™ scanner (CapitalBio Corp, Peking, China).

Intensity data was extracted from the TIFF images using LuxScan™ 3.0 software (CapitalBio Corp, Peking, China). The low-intensity spots in which fewer than 30% of the signal pixels exceeded the median background plus two times its standard deviation were removed. Then normalization was performed based on mean array intensity for inter-array comparison. To determine the significant differentially expressed miRNAs, Significance Analysis of Microarrays (SAM, version 3.0, from Stanford University) was performed using a two class unpaired comparison in the SAM procedure [14]. miRNAs with adjusted P values less than 0.05 and with either a greater than 2.0-fold change or less than a 0.5-fold fold change in expression compared with controls were identified as differentially regulated miRNAs[15,16]. The entire datasets (for miRNAs) described here are available from the Gene Expression Omnibus(GEO, http://www.ncbi.nlm.nih.gov/geo/) through series accession number GSE19695.

### Quantitative reverse trancriptase-polymerase chain reaction (qRT-PCR)

To validate the microarray results in this study, four of the de-regulated miRNAs were randomly selected for further validation by qRT-PCR.

Since miRNAs are ~ 22 nucleotides in length, it could not be reverse transcribed according to traditional method. So we designed a stem-loop RT primer to induce a reverse transcribe reaction. For PCR reaction, Gene-specific anti-sense primers and a universal sense primer were designed according to miRNAs sequences. All primers used in this study were synthesized at Invitrogen (Invitrogen, Peking, China). All the primer sequences were listed in an additional file (see Additional file 1).

Each reaction mixture of RT contained RNA samples including about 100 ng of purified total RNA, 50 nM stem-loop RT primer, 1× RT buffer, 0.25 mM each of dNTPs(Takara), 0.1 mM DTT, 200 U Superscript II reverse transcriptase (Invitrogen), and 20 U RNase inhibitor(Takara). The 20 uL reactions were incubated for 30 min at 16°C, 30 min at 42°C, 5 min at 85°C, and then held at 4°C[17].

Quantitative Real-Time PCR was performed using the Roche 1.2 real-time PCR instrument (Roche). The expression of the U6 small nucleolar RNA gene was used as an internal control.

The PCR was performed in triplicate, the 20 uL PCR reaction contained: 1 μL of cDNA solution, 2 uL LightCycler-FastStart DNA Master SYBR Green I (Roche), and 0.3 uM of each primer. The reactions were incubated at 95°C for 10 min, followed by 40 cycles of 95°C for 15 s, 60°C for 30 s. Samples were also run on a 1.5% agarose gel to confirm specificity.

The relative expression level for each miRNA was calculated using the comparative CT method [18,19]. To account for possible differences in the amount of starting RNA, miRNA expression was normalized to small nucleolar RNA U6.

### Transcription factors (TFs) and targets of miRNAs

The transcription of miRNAs is regulated by TFs and miRNAs exert their functions by regulating target mRNAs. Therefore, the TFs and targets of miRNAs may provide clues to the functions of miRNAs. We obtained experimentally validated miRNA targets from TarBase [20]. As a result, 9 of the 17 deregulated miRNAs having one or more targets were found. In total, 195 miRNA-target pairs were identified (see Additional File 2). We obtained the TFs that regulate the 17 deregulated miRNAs from TransmiR[21]. As a result, we identified 14 experimentally validated TF-miRNA pairs, in which 14 unique TFs regulate 6 of the 17 deregulated miRNAs (see Additional File 3).

### Gene function enrichment analysis

We carried out gene function enrichment analysis for miRNA transcription factors, miRNA targets, and miRNA host genes using EASE[22]. We chose all human genes as the background gene list. Bonferroni corrections to P values were used in the analysis.

## Results

### Altered expression of miRNAs in the myocardium of rats with AMI

**Figure 1:**
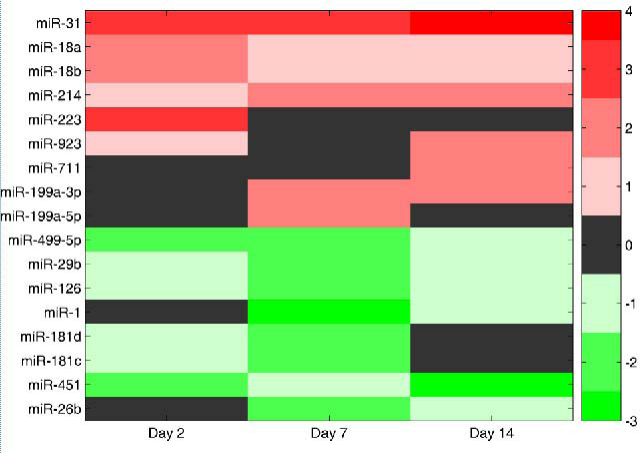
**A heatmap of deregulated miRNAs in the myocardium of rats with AMI for three time points**. A log-squared value of the fold change was imaged. Fold change values that were not available were imaged as a black color.

Microarray data mining and differential analyses resulted in 17 significantly deregulated miRNAs associated with AMI (Table 1, Figure 1). On day 2, miR-31, miR-223, miR-18a, and miR-18b were up-regulated, whereas miR-451 and miR-499-5p were down-regulated. Two of these aberrantly expressed miRNAs (miR-223, mir-499) have been reported to be associated with inflammation [23-25]. On day 7, miR-31, miR-214, miR-199a-5p, and miR-199a-3p were up-regulated, whereas miR-181c, miR-29b, miR-26b, miR-181d, mir-126, mir-499-5p, and miR-1 were down-regulated. Some of the deregulated miRNAs (miR-181, miR-26, miR-1, mir-29, miR-214, miR-126, and miR-499) are reported to be related to hypoxia, cell development, and cell growth [1,5,7,25]. These results indicate that miRNAs may take part in the regulation of myocardial remodeling in the early stage of myocardial infarction. On day 14, miR-214, mir-923, miR-711, and miR-199a-3p, and miR-31 were up-regulated, of which miR-31 showed the most striking up-regulation (an increase of eight-fold compared with the sham-control). Among those seventeen deregulated miRNAs miR-31 was up-regulated on day 2, day 7 and day 14. miR-199a was up-regulated on day 7 and day 14. miR-214 was up-regulated on day 7 and day 14. miR-499 was down-regulated on day 2 and day 7.

**Table 1:** miRNAs differentially expressed in the myocardial tissues of rats with acute myocardial infarction.

miRNA	Tissues in which miRNA is most highly expressed tissue	Day 2 (Fold change/Q-value)	Day 7 (Fold change/Q-value)	Day 14 (Fold change/Q-value)
miR-31	Colon	4.504/0.000^#^	5.923/0.000^#^	8.224/0.000^#^
miR-18a	Small intestine	2.136/0.013^#^	1.016/0.507	0.904/0.612
miR-18b	Small intestine	2.045/0.000^#^	1.502/0.023	1.314/0.000
miR-214	Distal colon	1.970/0.000	2.992/0.000^#^	2.404/0.000^#^
miR-223	Spleen	4.063/0.013^#^	n/a	n/a
miR-923	n/a	1.461/0.160	n/a	2.232/0.000^#^
miR-711	n/a	n/a	n/a	2.542/0.000^#^
miR-199a-3p	Pericardium	n/a	2.536/0.016^#^	3.321/0.000^#^
miR-199a-5p	Pericardium	n/a	3.236/0.000^#^	n/a
miR-499-5p	Right ventricle	0.435/0.027^┼^	0.288/0.016^┼^	0.889/0.612
miR-29b	Skeletal muscle	0.732/0.027	0.435/0.016^┼^	0.861/0.612
miR-126	Right ventricle	0.580/0.027	0.351/0.016^┼^	0.779/0.612
miR-1	Skeletal muscle	n/a	0.193/0.000^┼^	0.612/0.480
miR-181d	Brain	0.593/0.027	0.392^┼^	n/a
miR-181c	Brain	0.545/0.027	0.447^┼^	n/a
miR-451	Bladder	0.499/0.032^┼^	0.782	1.186
miR-26b	Placenta	n/a	0.419^┼^	0.878

The results show fold change between experimental samples and sham-control samples at three time points.

n/a: means that the corresponding data is not available.

#: significantly up-regulated miRNAs with fold change ≥ 2.0 and Q ≤ 0.05.

┼: significantly down-regulated miRNAs with fold change ≥ 0.5 and Q ≤ 0.05.

**Figure 2:**
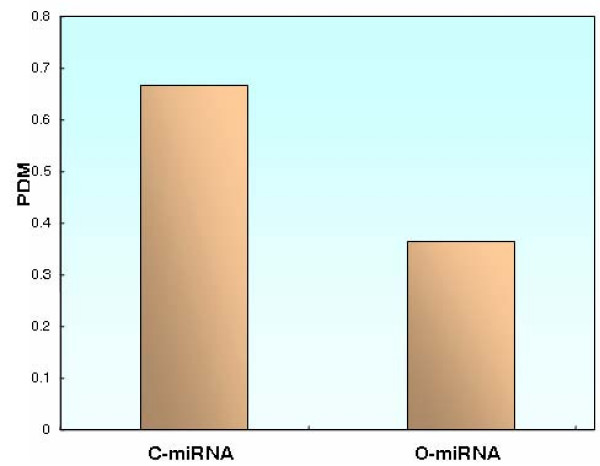
**Deregulations of C-miRNAs and O-miRNAs**. miRNAs show higher expression levels in normal cardiovascular tissues (C-miRNAs) tend to have higher probability of down-regulation in AMI compared with other miRNAs (O-miRNAs). "PDM" denotes the percentage of down-regulated miRNAs.

For the 17 deregulated miRNAs, the down-regulated miRNAs tended to be more enriched in miRNAs which are highly expressed in normal cardiovascular tissues, whereas the up-regulated miRNAs tend to show lower levels of expression in normal cardiovascular tissues. We investigated miRNA expression in normal tissues based on miRNA expression data provided by Liang et al. [26]. As a result, among the 17 deregulated miRNAs, six of them (C-miRNAs) showed the highest expression level in cardiovascular tissues, and the other 11 miRNAs showed the highest expression level in non-cardiovascular tissues (O-miRNAs) (Table 1). Furthermore, 66.7% (4/6) of the C-miRNAs were down-regulated, whereas only 36.4% (4/11) of the O-miRNAs were down-regulated (Figure 2). Therefore, C-miRNAs tend to be down-regulated in AMI, although the statistical test was not significant (P = 0.25, Fisher's exact test), owing possibly to small sample size.

### Confirmation of the altered expression of miRNAs by qRT-PCR

**Figure 3:**
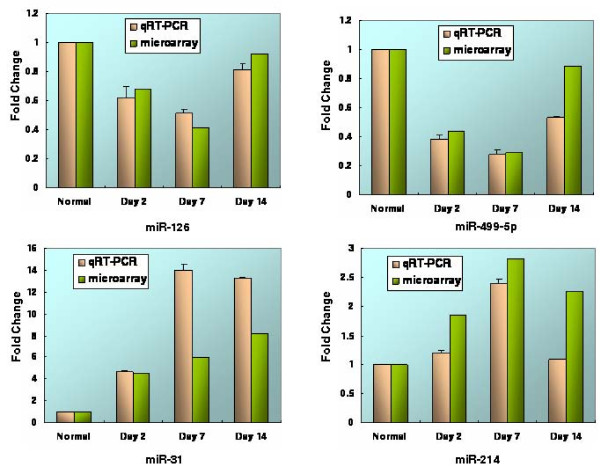
**A comparison of fold change based on qRT-PCR and microarray analysis for four miRNAs**.

To verify the accuracy of the microarray results above, we randomly selected four of the seventeen deregulated miRNAs for further confirmation using qRT-PCR. The result of qRT-PCR was consistent with that of microarray analysis (Figure 3 and Additional File 4). Expression levels of miR-126 and miR-499 were greatly down-regulated after AMI, whereas expression levels of miR-31 and miR-214 increased after AMI (Figure 3 and Additional File 4).

### Enriched functions of TFs and targets of miRNAs

We analyzed using EASE the enriched functions of TFs that were regulating the 17 deregulated miRNAs and the enriched functions of the targets of the 17 deregulated miRNAs [22]. As a result, both sets of genes were enriched in cardiovascular-associated functions (see Additional File 5 and File 6). For example, the significant gene functions for miRNA TFs include "myogenesis" (Bonferroni P = 1.59 × 10^-4^) and "muscle development" (Bonferroni P = 9.21 × 10^-4^); the significant gene functions for miRNA targets include "muscle thin filament tropomyosin" (Bonferroni P = 5.93 × 10^-3^) and "striated muscle thin filament" (Bonferroni P = 5.85 × 10^-2^). This result indicates that the deregulated miRNAs tend to have roles in cardiovascular disease by coupling with cardiovascular function-associated TFs and target genes.

miRNAs are reported to be non-randomly distributed in the genome. For example, many miRNAs (intronic miRNAs) are located in the introns of protein-coding genes (host genes) and show high co-expression with the host genes[27], suggesting the intronic miRNAs may have associated functions with their host genes. Therefore, we analyzed the functions of the host genes of the 17 deregulated miRNAs. As a result, seven of the seventeen deregulated miRNAs are located in the introns of protein-coding genes (Table 2). The function enrichment analysis of the host genes did not find enriched functions. This may be the result of a small sample size of only seven host genes. We next manually curated the functions of these host genes and found that most host genes (6/7) were associated with cardiovascular-related functions (Table 2), suggesting that the deregulated miRNA may have functions in ischemia myocardium by co-expression with their host genes. In conclusion, the gene function enrichment analysis provided further evidence that the deregulated miRNAs we identified were associated with AMI.

**Table 2:** Deregulated miRNAs that are located in the introns of protein-coding genes and host genes as well as the functions of host genes.

miRNA	Host gene	Function of host gene
miR-923	UNC45B	UNC45B plays a role in myoblast fusion and sarcomere organization
miR-126	EGFL7	blood vessel development; angiogenesis; and vasculogenesis
miR-26b	CTDSP1	n/a
miR-199a	DNM2/DNM3	filopodium formation; centronuclear myopathy; growth and development of megakaryocytes
miR-214	DNM3	filopodium formation; centronuclear myopathy; growth and development of megakaryocytes
miR-499	MYH7B	cardiac muscle, striated muscle contraction, striated muscle thick filament

## Discussion

miRNA regulates gene expression by binding and modulating the translation of specific miRNAs. In this study, we identified 17 deregulated miRNAs in a rat model of AMI. Our data shows that the change of expression levels of miRNAs is in accordance with the progression of AMI. In the early stage of myocardial infarction, the pathological change in the myocardium occurs with inflammation and intracellular and interstitial myocardial edema. Other pathological changes such as hypoxia, myocardial necrosis, fibrosis are secondary. Some deregulated miRNAs that respond to AMI are reported to be associated with hypoxia, cell differentiation, inflammation, development, and fibrosis [1,5,7,23,25]. These results suggest that miRNAs have a critical role in cardiac remodeling after AMI. Furthermore, miRNAs highly expressed in cardiovascular tissues tend to be down-regulated after AMI, indicating that a high level of expression is needed for these miRNAs to maintain the normal functions of the cardiovascular system. This suggests that miRNAs may have important roles not only in the normal development of the cardiovascular system but also in cardiovascular diseases.

Finally, function enrichment analyses was carried out upon TFs, targets, and host genes of the deregulated miRNAs. All these gene sets are enriched in cardiovascular functions, providing more solid evidence of the associations between these deregulated miRNAs and acute myocardial infarction. Although it is still unclear how these deregulated miRNAs exert their effects in the progression of myocardial infarction.

## Conclusion

In conclusion, we identified some miRNAs that showed altered expressions in rat myocardium with acute myocardial infarction. These miRNAs may be potential biomarkers for the diagnosis and treatment of acute myocardial infarction. Furthermore, investigating how these miRNAs exert their effects in acute myocardial infarction may become increasingly important.

## Competing interests

The authors declare that they have no competing interests.

## Authors' contributions

WG designed the whole study, JW designed the analysis of bioinformatics, BS and YG performed the experiment, BS and JW wrote the paper. All authors read and approved the final manuscript.

## Pre-publication history

The pre-publication history for this paper can be accessed here:

http://www.biomedcentral.com/1471-2261/10/11/prepub

## Supplementary Material

Additional file 1 Primers for qRT-PCR reaction. Click here for file

Additional file 2 Data on miR-target from Tarbase and miR-target from literature. Click here for file

Additional file 3 Relationship between TFs and deregulated miRNAs in rat myocardium with AMI. Click here for file

Additional file 4 Analysis of microarray and qRT-PCR of four deregulated miRNAs in rat myocardium with AMI. Click here for file

Additional file 5 Analysis of enriched function of TFs which are regulating the 17 deregulated miRNAs in rat myocardium with AMI and enriched function of targets of the 17 miRNAs. Click here for file

Additional file 6 Results of Analysis of enriched function of TFs which are regulating the 17 deregulated miRNAs in rat myocardium with AMI and enriched function of targets of the 17 miRNAs. Click here for file
